# Delayed LY333013 (Oral) and LY315920 (Intravenous) Reverse Severe Neurotoxicity and Rescue Juvenile Pigs from Lethal Doses of *Micrurus fulvius* (Eastern Coral Snake) Venom

**DOI:** 10.3390/toxins10110479

**Published:** 2018-11-17

**Authors:** Matthew R. Lewin, Lyndi L. Gilliam, John Gilliam, Stephen P. Samuel, Tommaso C. Bulfone, Philip E. Bickler, José María Gutiérrez

**Affiliations:** 1 Ophirex, Inc., Corte Madera, CA 94925, USA; tommaso.bulfone@gmail.com; 2 California Academy of Sciences, San Francisco, CA 94118, USA; paulshania@yahoo.co.uk; 3 Department of Veterinary Clinical Sciences, Center for Veterinary Health Sciences, Oklahoma State University, Stillwater, OK 74078, USA; l.gilliam@okstate.edu (L.L.G.); john.gilliam@okstate.edu (J.G.); 4 Queen Elizabeth Hospital, Kings Lynn, Norfolk PE30 4ET, UK; 5 Anesthesia and Perioperative Care, University of California, San Francisco, CA 94143, USA; bicklerp@anesthesia.ucsf.edu; 6 Instituto Clodomiro Picado, Facultad de Microbiología, Universidad de Costa Rica, San José 11501-2060, Costa Rica; jose.gutierrez@ucr.ac.cr

**Keywords:** snakebite, envenoming, antidote, inhibitor, coral snake, *Micrurus fulvius*, PLA2, phospholipase A2, neurotoxicity, antivenom

## Abstract

There is a clear, unmet need for effective, lightweight, shelf-stable and economical snakebite envenoming therapies that can be given rapidly after the time of a snake’s bite and as adjuncts to antivenom therapies in the hospital setting. The sPLA2 inhibitor, LY315920, and its orally bioavailable prodrug, LY333013, demonstrate surprising efficacy and have the characteristics of an antidote with potential for both field and hospital use. The efficacy of the active pharmaceutical ingredient (LY315920) and its prodrug (LY333013) to treat experimental, lethal envenoming by *Micrurus fulvius* (Eastern coral snake) venom was tested using a porcine model. Inhibitors were administered by either intravenous or oral routes at different time intervals after venom injection. In some experiments, antivenom was also administered alone or in conjunction with LY333013. 14 of 14 animals (100%) receiving either LY315920 (intravenous) and/or LY333013 (oral) survived to the 120 h endpoint despite, in some protocols, the presence of severe neurotoxic signs. The study drugs demonstrated the ability to treat, rescue, and re-rescue animals with advanced manifestations of envenoming. Low molecular mass sPLA2 inhibitors were highly effective in preventing lethality following experimental envenoming by *M. fulvius*. These findings suggest the plausibility of a new therapeutic approach to snakebite envenoming, in this example, for the treatment of a coral snake species for which there are limitations in the availability of effective antivenom.

## 1. Introduction

Snakebite envenoming is a significant global health concern and has been recently included in the World Health Organization (WHO) list of Neglected Tropical Diseases [1]. Over 5 million people are affected every year by snakebites, and over 75% of the estimated 138,000 deaths occur outside of the hospital setting, where antivenom cannot be administered [2,3,4]. In total, there are an estimated 5.8 billion people worldwide living within the geographic range of venomous snakes. A disproportionate number of impoverished populations reside in hot-spots where there is a high risk of receiving a snakebite and are remote from hospital resources and antivenom [3]. Worldwide, snakebite envenoming is an occupational risk to the majority of people working in manually dominated agricultural settings [5,6]. There is a clear, unmet need for effective, heat stable, and economical broad-spectrum snakebite envenoming therapies based on drugs with a high volume of distribution that can be given rapidly and at the time of bite as well as an adjunct therapy to improve the efficacy of antivenoms [7,8].

All snake venoms contain multiple enzymatic and non-enzymatic components, including abundant phospholipases A_2_ (PLA2), which are major contributors to venom toxicity of the majority of the world’s venomous snakes [9,10]. Snake venom secretory sPLA2 and sPLA2-like proteins play a fundamental role in neuromuscular paralysis, coagulopathy, cardiotoxicity, renal toxicity, and skeletal muscle necrosis that can result in rapid death or permanent disability [11,12,13]. Furthermore, there is an increasing body of evidence that mammalian homologs of venom sPLA2 also play a major contributory role in the endogenous inflammatory response, which might be involved in the response to snake venoms, including hemolysis, platelet dysfunction, and possibly fibrinogen depletion [14,15,16,17,18]. It is possible that the combined effects of venom and endogenous PLA2s has a critical role in the toxic inflammatory response, morbidity, and mortality resulting from snakebite envenoming.

Because of the central role played by snake venom sPLA2s, they are a logical target for therapeutics with active and peripheral site targets not accessible to antivenom therapies, owing to pharmacokinetic considerations and to the generally low antigenicity of PLA2s [19,20]. As a class of therapeutics, some limitations inherent to antivenoms (e.g., poor tissue penetration with a dependence on contact with circulating venom) are compounded by their perishable nature and the development of adverse reactions in a percentage of patients; thus, antivenoms have to be administered in health facilities by medical personnel [21].

By focusing on the enzymatic activity of sPLA2s, rather than their antigenicity, we have identified what could be a safe, potent set of compounds that can be readily re-purposed to timely address the cardinal life-threatening complications of snakebite envenoming in the field. LY315920 was first developed as a potent inhibitor of human sPLA2 for indications such as pancreatitis, sepsis, and cardiovascular disease [22]. Surprisingly, LY315920 and closely related compounds such as the orally-available pro-drug LY333013, are extremely potent inhibitors of 28 medically important venom sPLA2s from snake species of six continents [7]. These results have been replicated with several venoms both in vitro and in vivo in mice [7,23,24]. Both lead compounds have been through extensive human and animal testing, and although they have excellent safety profiles for short-term use, have never gained FDA approval and are now off patent [22,25]. Recently, we demonstrated that LY333013 given orally could rescue mice with treatment delays at time points past which specific Taipan (*Oxyuranus scutellatus*) antivenoms are effective [26]. LY315920 and its pro-drug LY333013, fit the profile of a low-cost, heat stable, easy to administer field antidote for snakebite envenoming, particularly for venoms whose main toxic actions are induced by PLA2s.

Herein, we show results from in vivo rescue studies with LY315920 and its pro-drug LY333013 in juvenile porcine models from lethal envenoming of *M. fulvius* venom. In animals, *M. fulvius* venom is known to cause neuromuscular paralysis, intravascular hemolysis, and myonecrosis [27,28,29]. *M. fulvius* venom was chosen for porcine testing because of its clinical relevance in North America, the fact that there is a scarcity of available antivenoms, and because its main toxic activities depend on the action of PLA2s [27]. In addition, *M. fulvius* venom proved to have high reliability in preliminary porcine lethality tests, reducing the likelihood of unnecessary animal use [30,31,32,33].

## 2. Results

**Figure 1:**
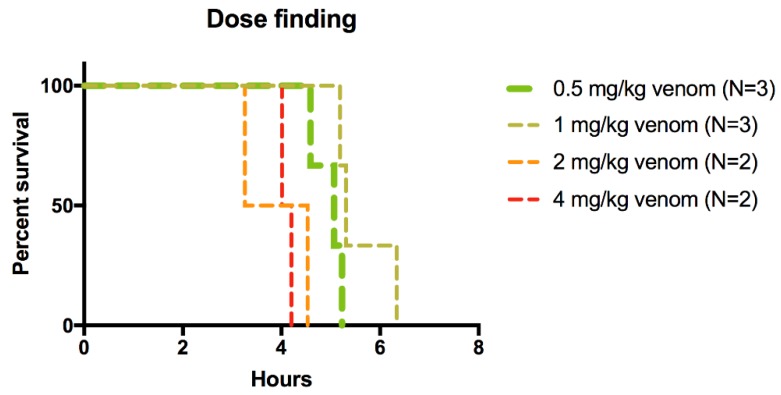
Kaplan Meier curves of lethality in pigs receiving various doses of *M. fulvius* venom.

Subcutaneous administration of 0.5–4.0 mg/kg *M. fulvius* venom killed all pigs within 6.5 h, as shown in Figure 1. Given the similarity of the effects of these doses on survival, doses of 1 mg/kg and 0.5 mg/kg were ultimately used but only one of these doses was used in any experiment and in appropriate control/treatment pairs. The venom doses used correspond to the maximum plausible quantity of venom that could be delivered by the bite of a large Eastern coral snake [34]. The group receiving a 0.5 mg/kg venom dose had a mean survival time of 4 h 48 min (S.D. = 20.53 min). All animals treated with LY315920 and/or LY333013 survived regardless of protocol (N = 14). In two animals, attempts were made to assess whether the neurotoxic effect was due to alpha-neurotoxins acting at the cholinergic receptor of the motor end-plate of muscle fibers; a combination of edrophonium (1 mg/kg) and atropine (0.02 mg/kg) combined in the same syringe was administered slowly intravenously over one minute in two animals receiving a lethal dose of the venom. No clinically observable improvement in strength occurred, suggesting, but not proving, the absence of any contribution by post-synaptically acting alpha-neurotoxins [35,36]. Notably, no animal in the study required any additional analgesia more than the mandatory two initial doses required by protocol (see Materials and Methods Detail, Section 5). 

### 2.1. Rescue Experiments

**Figure 2:**
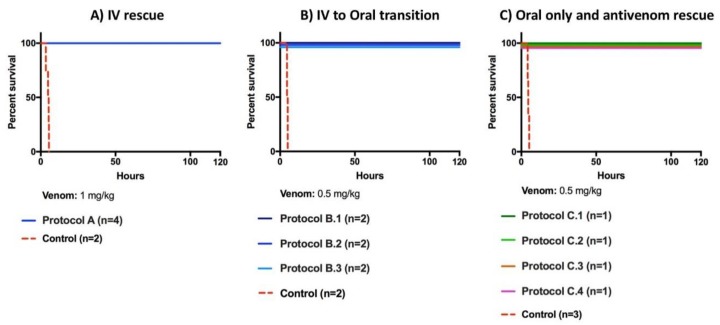
LY315920 (Intravenous) and LY3333013 (Oral) alone or in combination completely abrogated lethality induced by *M. fulvius* venom in different protocols: IV Rescue (**A**), IV to Oral transition (**B**) and Oral only and antivenom rescue (**C**) (see description of the various treatment protocols in the Materials and Methods section).

The first rescue study (Protocol A) produced 100% lethality in the control group within 5 h and 32 min (*n* = 2, 1 mg/kg venom and excipient) while all of the animals in the treatment group survived for the length of the study (*n* = 4, 1 mg/kg venom, 5 mg/kg bolus of LY315920 with 2.5 mg/kg bolus every 6 h and 1 mg/kg as needed after 24 h). Three out of four treatment pigs required only one 1 mg/kg LY315920 dose after the first 24 h, and only one pig required two doses, see Figure 2A.

In protocol “B”, combinations of intravenous (IV) continuous infusion and an oral drug were tested. All groups were injected with 0.5 mg/kg of venom, followed by various rescue protocols. The control group reached mortality within 5 h and 6 min (*n* = 2). All pigs in protocols B1, B2, and B3 survived to the end of the study period of 120 h, as shown in Figure 2B.

When the oral drug, LY333013, was compared to antivenom (protocol C), the control group reached mortality within 5 h and 20 min (*n* = 3). Again, all pigs submitted to the rescue protocol C survived the 120 h observation period, as shown in Figure 2C. Animals that where given antivenom within the first minutes of envenoming recovered, whereas those receiving antivenom after a 45 min delay suffered a delayed onset of clinical signs of severe envenoming. This delay caused the antivenom administration to fail when administered outside the time window during which it could be effective in neutralizing circulating venom. However, the pigs receiving a delayed administration of antivenom required fewer drug doses to reach the study endpoint, suggesting that the combination of drug and antivenom could have utility as a definitive treatment for snakebite even with significant delays in antivenom administration.

### 2.2. Recovery from Coagulopathy

**Figure 3:**
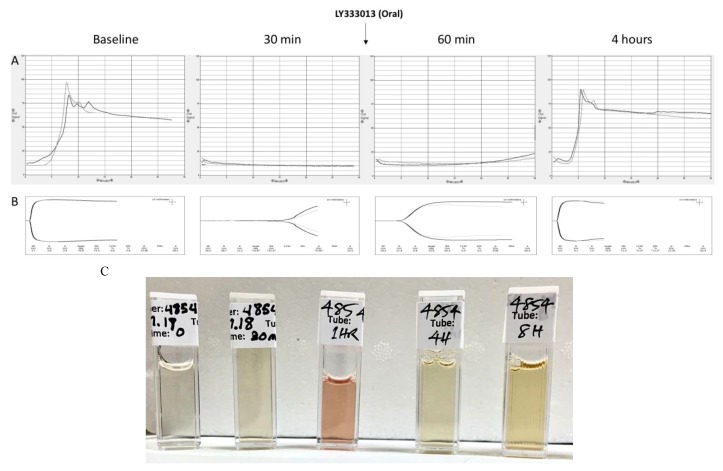
Evidence of systemic envenoming prior to initiation of treatment is illustrated by the incoagulability of blood. In this example, coagulopathy was corrected within a short time after oral administration of LY333013. Sonoclot (**A**) and Thromboelastography (TEG) (**B**). (**C**): A typical presentation of venom-induced gross intravascular hemolysis was frequently observed during the experiments. Samples correspond to plasma from pigs injected with 0.5 mg/kg venom and collected at times 0 min (1), 30 min (2), 1 h (3), 4 h (4) and 8 h (5). Notice the evident hemolysis in the sample at 1 h.

**Figure 4:**
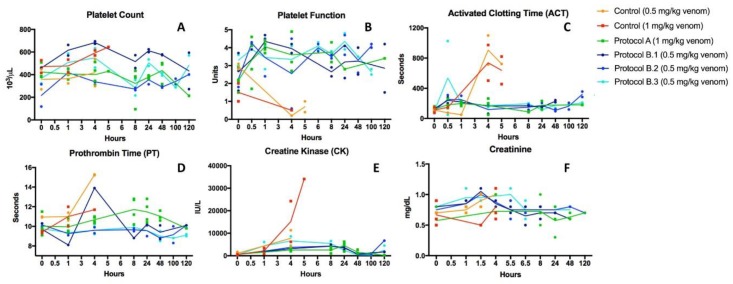
LY315920-treated animals showed a general preservation of hemostasis compared to controls. (**A**) Platelet counts remained normal but platelet function (**B**) was inhibited by venom. Platelet function was restored and maintained in the presence of drug. (**C**) Activated clotting time, (**D**) Prothrombin Time and (**E**) Creatine kinase largely remained within normal levels when animals were treated with sPLA2 inhibitors. Creatinine concentrations were normal for all subjects (**F**). Reference ranges: Platelet count 200–400 10^3^/µL, Platelet Function 2–4.8 Units, ACT 69–221 s, PT 10.5–13.5 s, CK 100–400 IU/L, Creatinine 0.1-2 mg/dL.

Thromboelastography (TEG) and Sonoclot outputs in basal samples, i.e., before the onset of envenoming, showed normal coagulation, as shown in Figure 3. Animals injected with venom developed a profound coagulopathy at 30 min, see Figure 3, as demonstrated by abnormal viscoelastic behavior. Treatment with oral LY333013 reverted coagulopathy in pigs, with partial correction by 60 min and complete recovery at 4 h. Likewise, control groups receiving venom only developed alterations in the Activated Clotting Time and Prothrombin Time, whereas envenomed pigs receiving the drug showed corrections in the values of these tests, approaching those of basal determinations, see Figure 4. *M. fulvius* venom did not affect the platelet numbers but did alter the platelet function, as shown in Figure 4. This alteration was corrected in the various protocols in which the drug was administered after envenoming, see Figure 4.

Laboratory samples at baseline and during the course of study were taken to assess the effect of the drugs in myotoxicity and renal parameters. As shown in Figure 4, envenoming resulted in a drastic increment in plasma creatine kinase (CK) levels, underscoring the development of myonecrosis. Envenomed animals treated with the drug under various protocols had much smaller increments in plasma CK activity, evidencing the inhibition of myotoxicity. No significant changes in plasma creatinine levels were observed in envenomed pigs, compared to basal values, thus indicating the lack of nephrotoxic action in this particular model of envenoming, see Figure 4. On the other hand, envenomed animals developed intravascular hemolysis, as evidenced by the presence of reddish pigmented plasma one hour after envenoming, see Figure 3C. This effect was abolished in the groups treated with the inhibitor (not shown). Myotoxicity and intravascular hemolysis in envenomings by *M. fulvius* in animal models are due to the action of venom PLA2s [37], which explains the beneficial effect of the drug.

### 2.3. Recovery from Neurotoxicity

**Figure 5:**
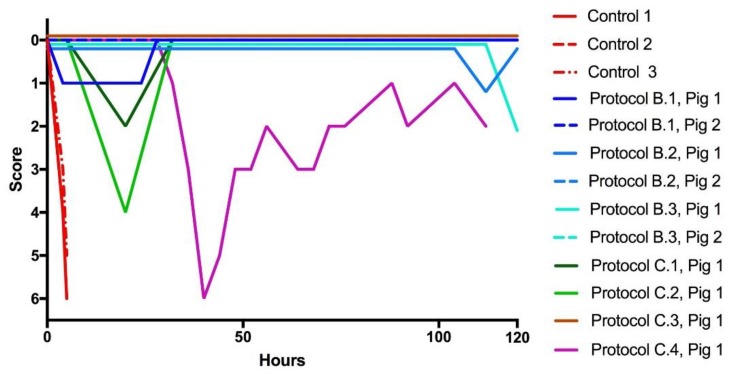
Clinical score in envenomed animals. All animals that received the test drug avoided or recovered from neurotoxicity. When rescue antivenom was not efficacious, the drug resolved severe neurotoxicity (Protocol C.4).

When the evolution of the clinical score was followed in envenomed pigs that did not receive any treatment, there was a rapid deterioration resulting in death within 5 and 6 h, see Figure 5. The majority of pigs treated with the drugs did not develop any sign of clinical deterioration and survived during the study period, as shown in Figure 5. In contrast, one pig in each of the rescue protocols B1, C1, and C2 showed an initial deterioration of clinical status, which then recovered, whereas pig 1 in protocol C4 showed a notorious clinical deterioration which could not recover after delayed administration of antivenom. Its condition clearly improved, and the animal survived, after a delayed oral administration of the drug, as shown in Figure 5.

### 2.4. Limitations

In toto, all animals treated with oral or IV drug survived, but there are only a small number of animals for each group; therefore, a comprehensive understanding of each protocol’s advantages or disadvantages is limited. Only three animals received antivenom (one each: immediately following envenoming and at 15 min and 45 min delays with decreasing efficacy over time). Future studies should be directed toward more a systematic dose finding for both antivenom and drug as they are likely to both be used in the clinical setting. For the purpose of this study, we stayed within LY333013 dose ranges and schedules previously tested in animals as well as Phase I and II human studies of these compounds that were previously evaluated for indications other than snakebite and never advanced to Food and Drug Administration (FDA) registration due to a lack of efficacy [22]. The results cannot yet be generalized to the neutralization of a broad range of snake venom sPLA2s and associated pathology but the toxin-specific approach is being increasingly investigated as a treatment for snakebite both alone and in conjunction with antivenom [7,9,10,23,24,26]. This was advantageous in guiding these experiments and to allow some reasonable comparison of results [30,31,32,33]. The domestic porcine model may be a stringent model to study gastrointestinal transit and absorption of drugs because gastric emptying in *Sus domesticus* is amongst the slowest of mammals and may have provided a more robust challenge to the oral drug, LY333013, than using minipigs bred for their accelerated gastrointestinal clearance [38]. All but one pig that was profoundly weak (Protocol C.4) were fed LY333013 in dog treats following recovery from anesthesia. Ensuring every bit of treat was consumed was difficult and if the treat became macerated it was challenging to get the pig to ingest every piece, thus some of the pigs may have received less than the full dose. One animal (Protocol B.3) required a new treat after refusing and spitting out the first offerings and likely received some additional drug for the first dose. In all animals where drug dosing was intermittent, neurological function showed waxing and waning consistent with the reported half-lives of the drugs. However, in all cases, where coagulopathy recurred or weakness reappeared, the drugs effectively reversed the clinical manifestations of envenoming within one to four hours. Animals cannot specifically express the subjective experience of pain, so the lameness score is a clinically used method used as a helpful guide for practitioners to quantify and communicate each case’s clinical signs. Using the system also creates a benchmark for monitoring the improvement or worsening of the disease. No animal in the study ever presented with isolated weakness of a limb—weakness was generalized and flaccid, while nociceptive type responses were typically manifested as a limp or forelimb withdrawal. We did not use electrophysiological methods to ascertain twitch responses or definitively eliminate the possibility of alpha-neurotoxin effects. This is because different alpha-neurotoxins show different affinities towards nicotinic cholinergic receptors (nAChR) of different animals. It may simply be that the high affinity of the alpha toxins towards the nAChR cannot be overcome by anticholinesterases. One straightforward method of showing this in other models is the isolated chick-biventer nerve-muscle preparation, which differentiates pre-synaptic from post-synaptic neurotoxicity [35,36]. However, this was not part of the present study, which was conducted on pigs. While the non-reversal of paralysis by acetylcholinesterase inhibitors (AChEIs) in pigs does not conclusively say that the paralysis was due to pre-synaptic neurotoxins, virtually all neurological symptoms (and coagulopathy) were rapidly abolished by the administration of sPLA2 inhibitors, but not AChEIs, suggesting sPLA2 toxicity was the main cause of weakness.

Finally, we used high doses of venom. It is possible that, using lower doses of venom, pigs could have been cured by fewer or lower drug doses. Venom sequestered in the fatty subcutaneous tissues of the pig could possibly be leaching out for days and require additional treatment. We subjected the drugs to much more stringent testing conditions than those reported in the literature and for what is required by industry and the World Health Organization (WHO) in the testing of antivenoms (which involve premixing of venom with antivenom prior to challenging animals) [39]. Our model is arguably more robust and amenable to making direct comparisons of therapeutics with different modes of delivery and mechanism and may argue for higher standards in the field as a whole [39].

## 3. Discussion

Continuous infusions or repeated bolus doses of LY315920 and oral LY333013 resulted in 100% survival following severe, experimental, *M. fulvius* envenoming in pigs. Rescue experiments with a single-dose of LY315920 produced a significant improvement in survival times compared to controls, but after 10+ h from the time of envenoming the symptoms often reappeared, probably due to a delayed venom absorption from a depot in the tissue site where venom was injected. In addition, this suggests that the half-life of the experimental drugs will make single dose therapy for severe envenoming unlikely to result in sustained clinical resolution. However, the ability to treat, rescue, and re-rescue animals at late stages of envenoming, including those already exhibiting severe neurological deficits and clotting disturbances, suggests these drugs might have unexpected versatility in a variety of clinical settings for both human and veterinary applications in *M. fulvius* envenoming.

We found that drug rescue where antivenom was initially unsuccessful resulted in a lowered drug requirement—i.e., fewer *pro re nata* (PRN) doses to maintain normal neurological function to the end of the study. This suggests that the circulating antivenom was able to capture drug-neutralized venom diffusing back into circulation from the peripheral tissues. However, confirmation of this requires more detailed studies.

PLA2s are predominant in *M. fulvius* venom and are largely responsible for its neurotoxicity [27]. Hence, in the dichotomic pattern described for *Micrurus* venoms from the proteomic standpoint, i.e., PLA2-rich and alpha-neurotoxin-rich venoms [40], *M. fulvius* fits within the first group. In addition, besides neurotoxicity (the primary toxicity in humans) other toxic activities associated with this venom are witnessed in different animal species (e.g., myotoxicity and intravascular hemolysis) and are also caused by the action of PLA2s [37]. This largely explains the success of these potent PLA2 inhibitors in abrogating lethality, myonecrosis, and hemolysis in our experiments in pigs. Moreover, PLA2s are known to affect coagulation and platelet function [13,41], hence explaining the ability of the inhibitors to abrogate these toxic effects as well. Our observations with edrophonium and atropine suggest that post-synaptically acting alpha-neurotoxins are not likely to play a significant role in this venom’s neurotoxicity, therefore supporting the concept that pre-synaptically acting PLA2s are the main neurotoxic components in *M. fulvius* venom. The key role played by PLA2s in the overall toxicity of *M. fulvius* venom makes it highly amenable to treatment with the PLA2 inhibitors tested in this study, a finding of potential impact in light of the current scarcity of coral snake antivenoms in the US.

Snakebite is an ancient scourge recognized in mythology and history since Biblical times but only recently as a neglected tropical disease by the WHO [1,42]. Our findings could represent a first crucial step away from the complete dependence on antivenom in combating the deadly effects of snakebite—especially, with the potential for developing an oral antidote to be taken at the time of a snake’s bite. Use of heat-stable, orally bioavailable, economical small molecules for initial and adjunct treatment for snakebite has the potential to substantially reduce mortality caused by snakebite in rural areas of the developing world. Additionally, this treatment could also reduce the mortality and morbidity associated with settings in which snakebite is an occupational hazard [5,6]. Further research is warranted on developing inhibitors to other enzymatic components of snake venom, such as metalloproteases [43]. Repositioning, the strategy utilized to repurpose LY315920 and LY333013 from their initial intended uses, offers the potential for low costs of development for future inhibitors. Combinations of multiple inhibitors and other antibodies could provide quite effective, safe, and affordable treatment for snakebite envenoming.

The inhibition of enzymatic activity of sPLA2s and other venom components by small molecule inhibitors of specific toxins could address many, but not all, limitations of antivenom. Notably, small molecule antidotes could potentially reduce the volume of antivenom administered, thus increasing the efficacy of antivenoms having a low potency against some venoms [8,21,43,44] while reducing the cost to the patient and to the healthcare system [45]. Recent US coral snake guidelines recommend availability of an antidote within one hour—the results of this paper suggest that LY315920 and LY333013 could fulfill these criteria. For example, neurologically intact patients could take the oral antidote while compromised patients could initially be treated with an IV formulation and transition to an oral therapy on recovery. In the US, there is currently no production or availability of previously approved coral snake antivenoms and these bites, while unusual, can be life-threatening, especially to children [46,47,48,49]. The 3-substituted indoles, such as LY315920 and/or LY333013, could offer a plausible, definitive treatment program for *M. fulvius* envenomed patients.

In conclusion, our findings suggest that LY315920 and/or LY333013 are likely to be effective in abrogating the main clinical manifestations in envenoming by *M. fulvius* and could apply to other types of envenoming as well [7,23,24,26]. Moreover, they support the concept that these drugs may potentiate the therapeutic action of coral snake antivenoms and others [26]. Owing to the current scarcity of *Micrurus* antivenoms in the US, the possibility of using these drugs in the management of these envenomings should lead to the design of clinical trials to assess their efficacy.

## 4. Materials and Methods

### 4.1. Venom

The venom of *M. fulvius* was purchased from Medtoxin Venom Laboratories (Deland, FL, United States of America (Lot 010918). Venom stock solutions (10 mg/mL) were prepared fresh daily in saline solution, and dilutions were performed in order to reach the desired venom dilutions to be injected.

### 4.2. Antidotes, Excipients, and Antivenom

LY315920 HCl (CAS 172732-42-5; ChemieTek, Indianapolis, Indiana) or LY333013 (CAS 172733-08-3; provided by Ophirex, Inc., Corte Madera, CA, USA) emulsions mixed into Greenies Hickory Smoke Flavored Pill Pockets (Mars Petcare, McLean, VA, USA) were prepared daily for each study. Control animals received excipients administered in exactly the same manner as the IV drugs. Boluses of LY315920 were administered manually over 15 min with continuous rate infusions (Vet/IV 2.2 Infusion Pump (Heska), Lubland, CO, USA) at a drug dosage of 0.67 mg/kg/hour according to the methods for previous Phase II human clinical trials for sepsis [50]. In the case of orally administered LY33301, the dose was 10 mg/kg (as was the dose administered to subjects in some human clinical safety trials) by offering the treats following emergence from anesthesia or by nasogastric feeding tube as an emulsion when animals were too weak to swallow. Antivenom: The monospecific Coral-ICP Antivenom produced at Instituto Clodomiro Picado (University of Costa Rica; batch number 5610615ACL) was used. It is a whole IgG preparation obtained by caprylic acid precipitation [51] from the plasma of horses immunized with the venom of *Micrurus nigrocinctus*. This antivenom is effective in the neutralization of *M. fulvius* venom [52].

### 4.3. Animals

A total of twenty-four mixed breed female juvenile *Sus domesticus* purchased through a USDA-pproved dealer were utilized in this study. The animals were anesthetized, catheterized, and euthanized according to methodology detailed in Materials and Methods Detail (Section 5).

### 4.4. Ethical Approval

The study and protocols were approved by the Institutional Animal Care and Use Committee at Oklahoma State University and per requirements of U.S. Federal Contract: W81XWH-17-C-0069, Ethical approval code: VM-17-31 Date of approval: 28 November 2017.

### 4.5. Study Period

The study period was 120 h from the time of experimental envenoming. Additional information regarding monitoring and euthanasia is included in Materials and Methods Detail (Section 5).

### 4.6. Envenoming and Rescue Experiment Protocols

Various doses of venom (0.5 mg/kg, 1.0 mg/kg, 2.0 mg/kg, and 4.0 mg/kg) were injected using a 26 gauge 0.5 inch needle in the distal lateral portion of the right antebrachium of the subject animals, see Table 1 and Table 2. The needle was inserted approximately 3 mm below the skin into the subcutaneous tissues and venom was injected. The time-course of deaths was followed in order to select a dose of venom for the remainder of the study. The same veterinarian (LLG) administered every dose of venom throughout the entire study period. Rescue experiments with study drugs or antivenom were carried out and several rescue protocols, as described in Table 2, were used after envenoming. Briefly, the ability of the sPLA2 inhibitors alone and in combination with antivenom were assessed in proof-of-concept efficacy experiments and in scenarios designed to potentially model clinical use alone or in combination with antivenom. IV drugs were administered either as boluses or by continuous rate infusion (CRI).

### 4.7. Clinical Assessment of the Severity of Envenoming

The evolution of the severity of clinical manifestations in envenomed animals was followed by the criteria indicated in Table 3. On occasion, animals favored the leg (suggestive of pain or tenderness) into which venom was injected. This was separately assessed by the lameness score for the purpose of pain management (see *Post-Envenoming Analgesia*).

### 4.8. Laboratory Tests

A venous blood sample was drawn through the catheter immediately following placement for baseline blood chemistry, complete blood count, coagulation panel tests (prothrombin time (PT), partial thromboplastin time, and dynamic viscoelastic coagulometry (TEG and Sonoclot^®^). More information on laboratory tests are detailed in the Materials and Methods Detail (Below).

## 5. Materials and Methods Detail

*Venom*: The venom of *M. fulvius* was purchased from Medtoxin Venom Laboratories (Deland, FL, USA) (Lot 010918). Venom stock solutions (10 mg/mL) were prepared fresh daily in saline solution, and dilutions were performed in order to reach the desired venom dilutions to be injected.

*Antidotes and Excipients*: LY315920 HCl (CAS 172732-42-5; ChemieTek, Indianapolis, Indiana, >99.9% purity by NMR, MS and HPLC) or an LY333013 (CAS 172733-08-3; provided by Ophirex, Inc. of Corte Madera, CA, USA) >99.9% pure by NMR, MS and HPLC) emulsion mixed into Greenies Pill Pockets (Mars Petcare, McLean, VA, USA) were prepared daily for each study and each subsequent day of the study. Briefly, LY315920 was weighed as a powder and mixed *w*/*w* in 1:1 sodium citrate and 1:2 mannitol followed by dissolution in 8.4% sodium bicarbonate to the final desired concentration of 5mg/mL LY315920. Particulates were filtered using 0.22 µm filters. Control animals received the intravenous (IV) sodium citrate/mannitol solutions without LY315290, and excipients were administered exactly as were the IV drugs. Boluses were administered manually over 15 min and continuous rate infusions (Vet/IV 2.2 Infusion Pump (Heska), Lubland, CO, USA) at 0.67 mg/kg/hour according to the methods for previous Phase II human clinical trials for sepsis [50]. The oral drug (LY333013) was prepared as a slurry as previously described [26]. Briefly, the drug was mixed with 8% *w*/*w* gum Arabic and then mixed into Greenies Pill Pockets. In one instance, the study animal became too weak to swallow on its own and the drug was administered via the orogastic route in gum Arabic alone. Study drugs were prepared by the study sponsor for intravenous administration and by sponsor or Oklahoma State University (OSU) veterinarians for oral administration studies when the sponsor was not present.

*Antivenom*: The monospecific Coral-ICP Antivenom produced at Instituto Clodomiro Picado (University of Costa Rica; batch number 5610615ACL) was used. It is prepared from the plasma of horses immunized with the venom of the coral snake, *M. nigrocinctus*. It is made of whole IgG molecules purified by caprylic acid precipitation [51]. The Median Effective Dose (ED_50_) of this antivenom against the venom of *M. nigrocinctus* is 0.4 mg venom/mL antivenom. This antivenom neutralizes the lethal and myotoxic activities of the venom of *M. fulvius* [52].

*Animals Detail*: Swine were housed in approved housing under a 12:12 light–dark cycle with ad libitum water and feed with the exception of an 8 h fast prior to anesthesia induction. All pigs were identified by ear tags. Pigs were acclimated to their new environment for 4–7 days prior to beginning the study. During this period, all animals were visually examined by trained laboratory personnel twice daily for any signs of illness. None of the pigs exhibited signs of illness prior to beginning the project. Pigs were weighed on the morning of the study and given a complete physical examination by Oklahoma State University animal husbandry staff (Control 12.72 ± 2.48 kg, treated 14.82 ± 2.28 kg (*p*-value: 0.18)). No animals were dropped from the study in either the control or experimental groups. All animals were fasted for a minimum of 8 h prior to anesthesia induction.

*Housing*: Swine were housed in pens (14 square feet) with raised plastic flooring to minimize contact with urine and feces. The pens allowed for individual or group housing depending on the phase of the study. During the acclimation period, pigs were group housed. Once intravenous catheters were placed, the pigs were individually housed to avoid the destruction of the catheters. Fresh water and feed were available at all times to the pigs during the studies with the exception of an 8-h fast prior to the day of catheter placement. Daily care of the pigs was provided by the centralized animal care unit, Animal Resources, as part of the Center for Veterinary Health Sciences’ animal care program accredited by AAALAC International. *Environment*: Swine were housed in a temperature-controlled environment with a 12/12 light–dark cycle. Animals were identified by ear tags provided by the USDA.

*Anesthesia, Instrumentation, and Monitoring*: Pigs were anesthetized using the following protocol: midazolam (5 mg/mL) was administered intramuscularly using a 20-gauge, 1-inch needle in the semimembranosus/semitendinosus region at a dose of 0.25 mg/kg. Pigs were allowed to rest quietly in their pen and become sedated for 15 min. Pigs were then anesthetized utilizing inhalant isoflurane given via an anesthetic mask. Once pigs were anesthetized they were endotracheally intubated and placed on maintenance isoflurane gas. Pigs were instrumented with a temperature probe and continuous cardiac rhythm monitoring (ECG). Heart rate, respiratory rate, and temperature were monitored every 15 min to measure anesthetic depth. Once the pigs were at an acceptable depth of anesthesia, intravenous and intra-arterial catheters were placed according to the following protocols.

*Intravenous catheter placement*: With pigs in dorsal recumbency, the front legs were retracted caudally until they were close to parallel with and secured to the surgery table. A triangle was visualized utilizing the caudal ramus of the mandible, the lateral manubrium and medial portion of the point of the shoulder and was sterilely prepared. Initially, catheters were blindly placed utilizing these landmarks and based on the procedure described by Fluornoy and Mani [53]. However, ultrasound guidance proved to increase the efficiency and safety of the procedure and so was employed. Briefly, a 18–5 mHz linear probe was placed inside a sterile glove to enable the use of ultrasound while maintaining sterility and the jugular vein was visualized. A 5 fr 13 cm double lumen central line catheter made by Arrow was inserted using standard Seldinger technique with the guide needle for the over the wire catheter was placed under ultrasound guidance. The catheter was then placed according to standard procedure over the wire catheter placement technique. Catheters were secured in place using 0 PDS suture and the catheters were wrapped with 4-inch Elastikon bandages.

*Intra-arterial catheter placement*: Arterial catheters (20 g, 1.88 in) were placed in the femoral artery and secured using 0 PDS suture, super glue, and 2-inch Elastikon bandages. Invasive blood pressure was measured using the arterial catheter every 15 min during anesthesia.

*Post-envenoming analgesia*: Analgesia (Buprenorphine 0.05 mg/kg IM) was given to all pigs prior to venom administration, at 4 h post envenoming, and then as needed every 4 h for a behavior indicating pain as judged by the attending veterinarian (e.g., limping or guarding of envenomed forelimb independent of overall neurological examination—e.g., generalized weakness clinically indicative of systemic neurotoxicity). For notation purposes, the assessment of pain was modified from the Obel laminitis grading system and the AAEP Lameness Scale.

*Euthanasia*: Pigs were euthanized or given rescue treatment if they reached a clinical score of 5 for two consecutive evaluations or if they reached a clinical score of 6 at any one evaluation. If they did not respond to rescue treatment they were humanely euthanized. All pigs were submitted for postmortem evaluation.

*Study Period Detail*: The pigs were monitored throughout the study period as follows:

Pigs were given a clinical score to record specific and general neurological status of the animals in a predetermined manner, see Table 3 of the manuscript and Figure 5 of results:

(1) Every 15 min post venom administration for the first 4 h following recovery from general anesthesia.
(2) From 4 h to 8 h post venom administration, pigs were given a clinical and lameness (veterinary surrogate for pain) score every 30 min.
(3) From 8 h to 48 h post venom administration pigs were given a clinical and lameness score every hour.
(4) From 48 h to 96 h pigs were given a clinical and lameness score every 6 h.

All monitoring was done in person until 48 h. After 48 h, if pigs were asymptomatic they were observed in person twice daily and monitored by video for the other time points. If pigs were noted to be showing signs of pain (lameness score of 3 or greater) or distress (down and unable to rise, dragging hind legs, visible signs of dyspnea such as open mouth breathing or abdominal press when breathing, or a clinical score of 4 or greater) one of the study veterinarians examined the pig in person within 1 h of noting abnormal signs. Pigs that experienced life-threatening toxicity defined as a clinical score of 5 for two consecutive observations or a clinical score of 6 once either received immediate treatment with the study drug or antivenom or were humanely euthanized using 39% sodium pentobarbital given intravenously.

In lethality-dose-finding studies, to deduce if the weakness was due to toxicity from alpha-neurotoxins which bind to the nicotinic cholinergic receptor at the motor end-plate, animals were challenged with edrophonium and atropine. Pigs were given a combination of edrophonium (1 mg/kg) and atropine (0.02 mg/kg) combined in the same syringe and given slowly intravenously over one minute and did not exhibit any changes in condition (*n* = 2 animals), thus indicating that neurotoxicity in this model is based on presynaptic activity of neurotoxins. This protocol was therefore discontinued.

Laboratory Test Details: An arterial blood sample was collected and assayed to measure PaO2 and PaCO2 during anesthesia at baseline and post venom just prior to recovery to ensure animals adequately oxygenated. There were no hypoxic episodes unrelated to envenoming and oxygenation/ventilation did not reverse venom-induced weakness (data not shown). Venous blood samples were drawn through the catheter immediately following placement for baseline blood chemistry, complete blood count, prothrombin time (PT), and dynamic viscoelastic coagulometry (TEG [54] and Sonoclot^®^). All laboratory testing, with the exception of the Sonoclot and TEG, was performed by Antech Diagnostics (Stillwater, OK, USA). In addition, after centrifugation of blood, the color appearance of plasma was observed in order to judge the presence of gross hemolysis. Sonoclot and TEG were performed bedside at OSU. Following blood draws, catheters were flushed with 3 mL of heparinized saline. Subsequent blood samples were collected at the following time points utilizing a two-syringe technique to avoid heparin contamination of the samples; 0, 30min, 60 min, 4 h, 8 h, 24 h, 48 h, 100 h, and 120 h [54]. Briefly, 3 mLs of blood were drawn from the catheter and discarded prior to drawing the sample. The sample was drawn and then the catheter was again flushed with 2 mLs of heparinized saline.

Sonoclot: Dynamic coagulation testing was utilized to demonstrate a more complete picture of the coagulopathy caused by *M. fulvius* envenoming. Sonoclot^®^ utilizes viscoelastic coagulometry to asses the time until clot formation, the strength of the clot that is formed, clot retraction, and clot lysis [55]. Values provided by Sonoclot^®^ testing are platelet function, activated clotting time (ACT = time to initiate fibrin formation), clot rate (rate at which fibrinogen is converted to fibrin), and time to peak (time to reach peak clot strength).

Thromboelastography (TEG): TEG 5000 Thromboelastograph Hemostasis System (Haemoscope Corporation, Niles, IL, USA) was used to collect TEG measurements. Citrated blood (1 mL) was transferred to a kaolin tube (Kaolin activator, Haemonetics Corporation, Braintree, MI, USA). A sample of kaolin-activated blood was transferred to a cup with calcium chloride. The TEG was initiated at 37 °C. Paired samples were taken from corresponding citrated blood and kaolin-activated tubes and then placed into two separate disposable cups. Several TEG parameters were collected, including R-time (or clotting time; normal values: 2–8 min), alpha angle (normal values: 55–788), maximal amplitude (normal values: 51–69 mm), and LY30 (normal <8%) [54]. An example of the technique compared to Sonoclot is shown as Figure 3B.

## Figures and Tables

**Table 1:** Lethality study in juvenile pigs injected with Eastern coral snake (*M. fulvius*) venom by the subcutaneous route (SC).

Group	Number Animals per Group	Venom Dose (mg/kg)
1	2	4 mg/kg
2	2	2 mg/kg
3	3	1 mg/kg
4	3	0.5 mg/kg

**Table 2:** Rescue study using LY315920 and LY333103 to treat juvenile pigs injected with Eastern coral snake venom (route: SC).

Protocol	Number Animals per Group	Treatment	Venom Dose Level	Drug (and/or Antivenom) Dose
Protocol A	4	Venom	1 mg/kg	-Loading dose: 5 mg/kg i.v. over 15 min. -2.5 mg/kg i.v. bolus q6 h × 24 h -1 mg/kg i.v. prn weakness/coagulopathy
		+		
		LY315920 (i.v.)		
Protocol B.1	2	Venom	0.5 mg/kg	-Loading dose: 1 mg/kg i.v. immediately -CRI i.v. 0.67 mg/kg/h × 24 h -Oral 200 mg q6 h × 4 then 100 mg PO q6 h prn weakness/coagulopathy
		+		
		LY315920 (i.v.) then LY333013 (p.o)		
Protocol B.2	2	Venom	0.5 mg/kg	-Loading dose: 1 mg/kg i.v. (15 min after envenoming) -CRI i.v. 0.67 mg/kg/h × 24 h -Oral loading dose of 400 mg PO 4 h prior to ending the CRI followed by 200 mg q6 h for 12 h then 100 mg PO q6 h prn weakness/coagulopathy
		+		
		LY315920 (i.v.) then LY333013 (p.o)		
Protocol B.3	2	Venom	0.5 mg/kg	-400 mg PO loading dose (15 min delay) -200 mg PO q6 h for 12 h -100 mg PO q6 h for 36 h -100 mg PO q6 h prn weakness/coagulopathy
		+		
		LY333013 (p.o)		
Protocol C.1	1	Venom	0.5 mg/kg	-46.4 mL antivenom i.v. (1 mL antivenom/0.125 mg venom) (15 min post-venom)
		+		
		Antivenom		
Protocol C.2	1	Venom	0.5 mg/kg	-67.2 mL antivenom i.v. (1 mL antivenom/0.125 mg venom) (>45 min post-venom) -67.2 mL antivenom i.v. prn weakness/coagulopathy (1 mL antivenom/0.125 mg venom) -Rescue with LY333013 200 mg PO × q6 h prn weakness/coagulopathy
		+		
		Antivenom		
		+		
		LY333013 (p.o)		
Protocol C.3	1	Venom	0.5 mg/kg	-200 mg PO (>45 min post-venom) -200 mg PO q6 24 h -100 mg PO q6 h for 24 h -200 mg PO weakness/coagulopathy
		+		
		LY333013 (p.o)		
Protocol C.4	1	Venom	0.5 mg/kg	-200 mg PO (>45 min delay) -200 mg PO q6 h for 12 h Drug withdrawn -Rescue with antivenom i.v. (1 mL/0.125 mg of venom) prn weakness/coagulopathy If no response to antivenom: -Rescue with 200 mg PO q6 h prn weakness/coagulopathy
		+		
		LY333013 (p.o)		
		+		
		Antivenom		

**Table 3:** Clinical score used in the assessment of the severity of envenoming.

Score	Clinical Picture
0	Normal activity, interested in food/water/toys, normal grunting, curious about environment, responds normally to stimulation by moving away, rises easily and quickly from recumbency when stimulated.
1	Normal movement around pen, reduced interest in food/water/toys, reduced interaction with environment or caretakers, rises from recumbency and moves away when stimulated.
2	Evidence of weakness in one or more limbs, reduced interest in food/water/toys, reduced responsiveness to stimulation but still able to rise normally from recumbency and remain standing >3 min.
3	Significant evidence of weakness (dog-like sitting, treading in back legs after rising) but able to rise unassisted when stimulated and stays standing >15 s but <3 min, some interest in food/water/toys.
4	Significant evidence of weakness (dog-like sitting, sternal or lateral, treading in back legs after rising), requires assistance to rise but can stand longer than 10 s on their own once assisted to stand.
5	Significant evidence of weakness (dog-like sitting, sternal or lateral, treading in back legs after rising), unable to rise without assistance, remains standing <10 s.
6	Marked evidence of weakness (sternal or lateral recumbency) unable to rise even with assistance. Meets criteria for Euthanasia
